# Performance of Saliva Samples for COVID-19 Diagnosis by Using the Allplex^TM^ 2019-nCoV Assay Kit

**DOI:** 10.3389/fmed.2021.617399

**Published:** 2021-02-24

**Authors:** Cecilia V. Tapia, Campos Marcia, Mora Ivone, Pozas Nadia, Morales Lesly, Guzmán Camila, Aguilera Valentina, Ibarra Paula, Magne Fabien

**Affiliations:** ^1^Laboratorio de Especialidad Clínica Dávila, Santiago, Chile; ^2^Alatheia Medical Company, Santiago, Chile; ^3^Programa de Microbiología y Micología, Facultad de Medicina, Instituto de Ciencias Biomédicas, Universidad de Chile, Santiago, Chile

**Keywords:** COVID-19, RT-qPCR, nasopharangeal swabs, SAR-CoV-2, saliva

## Abstract

**Background:** Although the nasopharyngeal swab (NPS) is the reference sampling method for the detection of SARS-Cov-2, it is not always possible to collect NPS in some patients. Saliva represents an interesting sampling method because it is less invasive and more convenient in patients with nasal or pharyngeal lesions.

**Objective:** To compare the RT-qPCR test performances of saliva samples with nasal mid-turbinate swab (NMTS) and NPS samples in a cohort of ambulatory patients suspected of having COVID-19.

**Study Design:** For each of the 112 enrolled patients, NPS, NMTS, and saliva samples were collected and tested for SARS-Cov-2 detection using three different target genes (RdRP, N and E genes) by RT-qPCR.

**Results:** Among the positive samples (56/112), saliva samples showed a lower percentage of SARS-Cov-2 detection compared to NPS samples, (85.7 *vs*. 96.4%), while still a lower percentage was observed for NMTS samples (78.6%). In average, saliva samples showed higher Ct values for all tested target genes, compared to those from NPS and NMTS samples.

**Conclusions:** By using the Allplex^TM^ 2019-nCoV Assay Kit, saliva samples showed lower sensitivity for SARS CoV-2 compared to NPS samples; however, the not detected cases had lower viral burden in NPS samples (CT values >33) representing an interesting alternative sampling method in patients in which it is not possible to take a NPS sample.

## Introduction

COVID-19 diagnosis is based on clinical, laboratory, and imaging features through computed tomography (CT) (1–3). Reference standard diagnostic tests for detection of SARS-Cov-2 in suspected infected patients includes reverse transcription polymerase chain reaction polymerase (RT-qPCR). To date, the Centers for Disease Control and Prevention (CDC) recommends nasopharyngeal swab (NPS) sampling for detecting viral ribonucleic acid of SARS-Cov-2 by RT-qPCR (2).

However, it is not possible to collect NPS in some patients, such as those with epistaxis or with an inflamed nasopharyngeal mucous membrane. Part of those patients must be regularly controlled with PCR to be able to go to work, and they suffer discomforts due to sampling. Also, this sampling method can constitute a risk in patients with thrombocytopenia and other coagulation disorders (1).

For these reasons, international technical organizations have approved alternative sampling methods, such as oropharyngeal swab (OPS) and nasal swabs (4, 5). Some studies reported similar accuracy between the nasal midturbinate swab (NMTS) and NPS sampling (4, 6). Additionally, nasal sampling presents several advantages, because it is less invasive, assumed to cause less discomfort, and can be moreover used to perform self-sampling.

In the same way, saliva samples also represent a promising sample because they are less invasive and more convenient for patients, compared to NPS (7–9). Saliva samples can be collected by patient themselves, minimizing then virus transmission to health care and reducing the use of personal protective equipment. Saliva contains a pool of microorganisms coming from the lower respiratory tract, nasopharynx, and infected salivary glands. In some coronaviruses infection, salivary glands were infected very early in the disease process (10). Based on these international studies, the Chilean Ministry of Health is evaluating to implement massively the collection of saliva for PCR diagnosis of COVID-19 in outpatient population. However, data to validate the use of saliva sampling for the detection of the SARS-Cov-2 is still lacking.

On the other hand, clinical diagnosis protocols based on PCR techniques must be previously validated and standardized for applying them on biological samples. Although commercial PCR kits are generally approved for use in patient diagnostics (the U.S. Food and Drug Administration or the “Conformitè Europëenne” marking), it is necessary to verify some parameters recommended by the manufacturer (11, 12). To date, all PCR detection kits for the COVID-19 diagnosis are validated only to collecting samples of NPS/OPS, sputum, and bronchioalveolar lavage. None of them is approved for saliva samples by either the manufacturer or the regulatory organisms.

Based on the previous facts, we compared the RT-qPCR test performance of the saliva sample with nasal and NPS samples in a cohort of ambulatory patients. For all RT-qPCR tests, we used the commercial Allplex^TM^ 2019-nCoV Assay Kit (Seegene, Korea), which was previously validated for clinical diagnosis with NPS, oropharyngeal swabs (OPS), sputum, and bronchioalveolar lavage (BAL).

## Materials and Methods

### Ethics Statement and Clinical Samples

One hundred and twelve patients, who attend to the Sample Collection Unit at Clínica Dávila (Santiago, Chile) for clinical diagnosis of COVID-19, were recruited. For each patient, three sample types were collected including NPS, NMTS, and saliva samples. NPS and NMTS samples were collected using a standard technique, as recommended by the manufacturer (Allplex^TM^ 2019-nCoV Assay insert), and transported to the laboratory in a viral transport medium that was prepared according to the standard operating procedure of the CDC in the United States (13). Saliva samples were collected through an assisted self-sampling; it was asked to patients to collect saliva by tilting the head back for 10s and then spit it into a sterile vial. A total of 1–2 mL of saliva per patient was collected and processed.

### RNA Extraction

Prior to RNA extraction, saliva samples were pre-treated by adding proteinase k and incubating at 56°C by 10 min based on the modified protocol of Chu et al. (14). Briefly, we eliminated the final heating at 98°C by 5 min step, to avoid possible effects on the viral load, as previously observed at high temperatures (15, 16). Otherwise, RNA extraction of all samples was performed using the STARmag kit (Seegene, Korea) following the manufacturer's instructions.

### RT–qPCR Conditions

Target gene amplification of SARS-Cov-2 was performed using the Allplex^TM^ 2019-nCoV Assay Kit (Seegene, Korea), according to the manufacturer‘s procedure. The qPCR preparation was carried out in the Starlet equipment (Hamilton, USA, distributed by Seegene) and the qPCR amplification in the CFX-96 thermocyclists (Biorad, USA). The Allplex^TM^ 2019-nCoV Assay Kit detects three viral genes (N, RdRP, and E). It was considered as inconclusive or indeterminate when the internal control was not amplified. It is currently considered as positive if both the N gene and RdRP were amplified. If only the E gene was amplified, it is considered as presumably positive, thus requiring repetition by another extraction instrument (in this case, MagNAPure Compact System, Roche).

### Statistics

ANOVA one-way multiple comparisons and *t*-student tests were performed for evaluating statistical difference between sampling methods using the GraphPrism 6.0 software.

## Results

### Clinical Data

One hundred and twelve patients, who were presenting COVID-19 symptoms, or being close contact of a COVID-19 patient, were included in this study. Prior to obtaining the samples, the patients signed an informed consent approved by the Ethics Committee of Clínica Dávila.

The median age was 37 years old with a range of 16–78 years, consisting of 39 females (34.8%) and 73 males (65.2%). The main symptoms at the time of the PCR were: headache (46.8%), myalgia (32.4%), cough (27.0%), fever (18.0%), odynophagia (9.0%), abdominal pain (7.2%), ageusia (7.2%), nasal congestion (5.4%), anosmia (5.4%), thorax pain (3.6%), asthenia (2.7%), dyspnea (1.8%). The onset of symptoms was on average 3 days prior to the consultation.

### Effect of the Sampling Method on the Sensitivity of the SARS-Cov-2 Detection

For all collected samples, the detection of SARS-Cov-2 was performed using three independent RT-qPCR methods, which target the amplification of three genes, including the envelope protein (E), the RNA-dependent RNA polymerase (RdRp), and the nucleocapsid protein (N) genes. In our study, the amplification of the RdRP and N genes were considered as a positive result, while the E gene amplification was a presumptive positive, according the manufacturer instructions. An exclusive amplification of the E gene was obtained in six samples (one NPS, two NMTS, and three saliva samples) from distinct patients, having Ct values close to 40. In these cases, we repeated the processing of these samples using another extraction method. For all these cases, the repeated analyses were negative, so we considered these samples as negative.

Of the total samples analyzed, 56/112 (50.0%) were SARS-Cov-2 positive for at least one of the three biological samples (RT-qPCR positive for the RdRP and N gene amplification). We did not observe the Ct mean difference between symptomatic and asymptomatic patients, although Ct values tend to be lower in symptomatic patients (data not shown). Among the SARS-Cov-2 positive samples (*n* = 56), 54/56 (96.4%) of NPS samples were positive, while 44/56 (78.6%) and 48/56 (85.7%) were positive in NMTS and saliva samples, respectively (Table 1). We found that 39/56 samples (64.2%) were positive for all the three sample types, 3/56 samples (5.4%) for both NPS and NMTS samples, and 5/56 samples (8.9%) for both NPS and saliva samples. On the other hand, 2/56 (3.6%) samples were positive in both NMTS and saliva samples.

**Table 1:** Prevalence of RT-qPCR positive samples for SARS-Cov-2 in the distinct sampling methods.

**NPS samples**	**NMTS samples**	**Saliva samples**	**n (% of total positive samples)**
Total SARS-Cov-2 positive samples (Positive samples for at least one of the three biological samples)			56 (100)
Positive	–	–	54 (96.4)
–	Positive	–	44 (78.6)
–	–	Positive	48 (85.7)
Positive	Positive	Negative	3 (5.4)
Positive	Negative	Positive	5 (8.9)
Negative	Positive	Positive	2 (3.6)
Positive	Negative	Negative	4 (7.1)
Negative	Positive	Negative	1 (1.8)
Negative	Negative	Positive	2 (3.6)

*The table represents the effective of positive samples according to the RT-qPCR results obtained for the distinct sampling methods. “NPS,” nasopharyngeal swab; “NMTS,” nasal mid-turbinate swab; “Positive,” SARS-Cov-2 RT-qPCR positive assay; “Negative,” SARS-Cov-2 RT-qPCR negative assay; “–” SARS-Cov-2 RT-qPCR assay not considered*.

### Effect of the Sampling Method on the Performance of RT-qPCR for SARS-Cov-2 Detection

**Figure 1:**
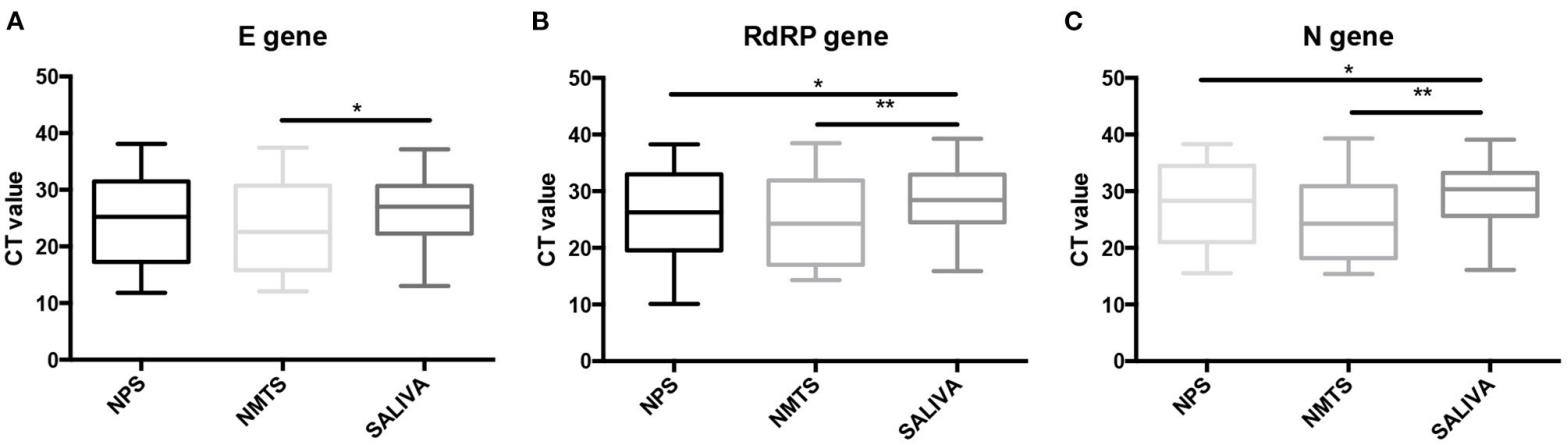
Comparison of the RT-qPCR performance between the distinct sampling methods. Boxplots of SARS-CoV-2 Cycle threshold (Ct) values (mean and interquartile range) obtained for **(A)** E target gene, **(B)** RdRP target gene, and **(C)** N target gene in nasopharyngeal swab (NPS), nasal mid-turbinate (NTMS) swab, and saliva (SALIVA) samples. Statistical differences were tested using the paired *t*-test. * *p* ≤ 0.05. ** *p* ≤ 0.01.

Next, we analyzed whether the sampling methods affect the performance of the SARS-Cov-2 detection. We compared the RT-qPCR performance between the distinct sample types for each of the three genes used for the detection of the SARS-Cov-2, including E, RdRp, and N genes. For these analyses, all samples were processed at the same time using the same RT-qPCR protocol (Figure 1). For the three target genes, the Ct values of nasal and saliva samples did not differ statistically from those of nasopharyngeal samples, although the Ct values of saliva samples tend to be higher for the three target genes. However, we found that the nasal and saliva samples showed difference in Ct values (Table 2). The Ct values of saliva samples were statistically higher than those of nasal samples (*p* = 0.0465 for E gene; *p* = 0.0087 for RdRP gene; and *p* = 0.0049 for N gene).

**Table 2:** Impact of sampling method on the RT-qPCR Ct values according to the target gene.

	**E gene**		**RdRP gene**		**N gene**	
**Sampling methods**	**Ct mean [95% CI]**	**Ct shift mean ±SEM (Ct_**x**_ -Ct_**NPS**_)**	**Ct mean [95% CI]**	**Ct shift mean ±SEM (Ct_**x**_ -Ct_**NPS**_)**	**Ct mean [95% CI]**	**Ct shift mean ± SEM (Ct_**x**_ -Ct_**NPS**_)**
NPS	24.47 [22.16–26.78]	–	26.06 [23.72–28.39]	–	27.54 [25.39–29.69]	–
NMTS	23.04 [20.48–25.61]	−0.86 ± 1.68	24.81 [22.32–27.29]	−0.53 ± 1.56	24.58 [22.26–26.89]	−2.38 ± 1.72
Saliva	26.58^*^ [24.66–28.50]	2.86 ± 1.60	28.66^*^ [26.8–30.51]	3.34 ± 1.37	29.27^*^ [27.45–31.09]	2.42 ± 1.18

“*NPS,” nasopharyngeal swab. “NMTS,” nasal mid-turbinate swab*.

* *significant difference with NPS (p < 0.05)*.

### Effect of the Sampling Method on the RT-qPCR Efficiency

**Figure 2:**
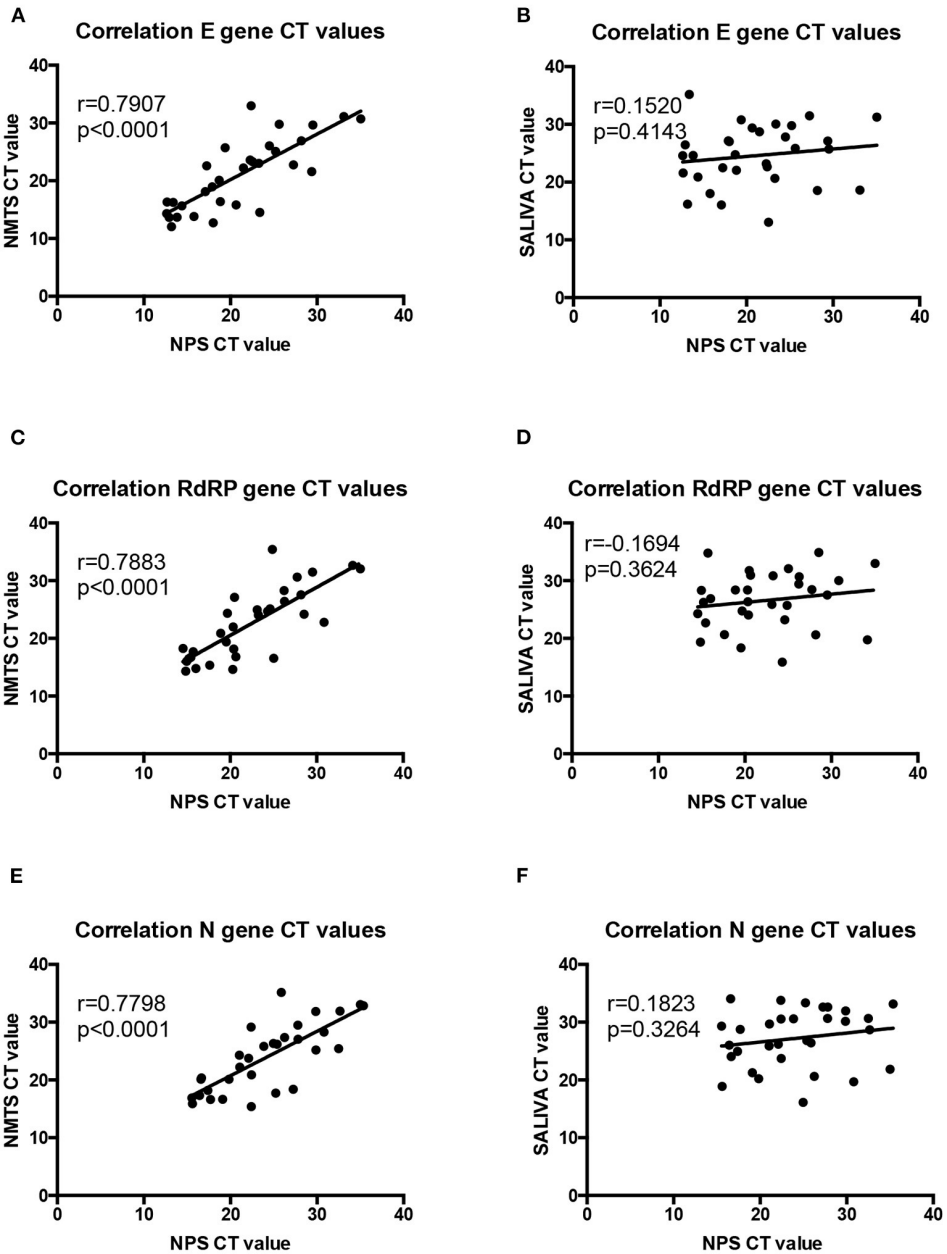
Comparison of the RT-qPCR efficiency between the distinct sampling methods. Correlation of SARS-CoV-2 Cycle threshold (Ct) values obtained for **(A,B)** E target gene, **(C,D)** RdRP target gene, **(E,F)** and N target gene between nasopharyngeal swab (NPS), nasal mid-turbinate swab (NTMS), and saliva (SALIVA) samples. NPS was considered as the sampling method reference.

For determining whether the sampling method influences the efficiency of RT-qPCR method used for SARS-Cov-2 detection, we compared the Ct values of samples obtained from nasal and saliva samples with those obtained from nasopharyngeal samples (Figure 2). We observed a positive correlation between the nasal samples and the nasopharyngeal samples for all three amplified-target gene-based PCR methods. However, Ct values obtained from the saliva samples did not correlate with those from nasopharyngeal samples for all three amplified-target gene-based PCR methods.

## Discussion

In this study, we provide a comparison of three sampling methods, including NPS, NMTS, and saliva samples, for detecting SARS-Cov-2. Compared to NPS samples, saliva samples showed a lower percentage of SARS-Cov-2 detection while an even lower percentage was observed for NMTS samples (Table 1). Also, the RT-qPCR-based detection method from saliva samples showed higher Ct values for all studied genes (E, RdRP, and N) compared to those from NPS and NMTS samples and a lower correlation with the NPS samples (Figures 2B,D,F) that could indicate a lesser analytical sensitivity with this sampling method.

For SARS-Cov-2 PCR detection, we used the Real-time PCR assay Allplex^TM^ 2019-nCoV Assay Kit, which previously showed a good clinical and analytical performance with PCR efficiency higher than 96% and has CE-IVD approbation (17). This point is important since the Real-time PCR Assay Kit that is clinically validated is generally not considered in studies. Because the manufacturer did not validate this kit for saliva samples, data of this study allowed clinically validating this kit for the COVID-19 diagnosis using this sample type.

**Figure 3:**
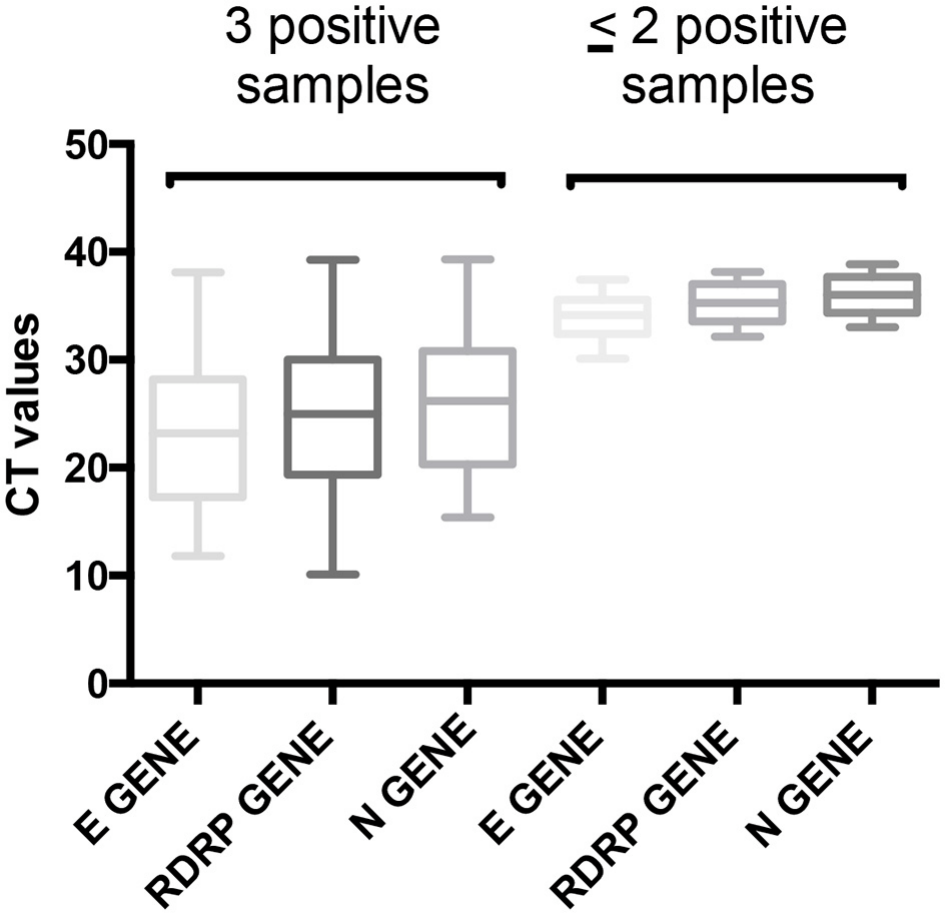
Comparison of Ct values depending on the number of positive samples. Ct values of samples that are positive for the three sampling methods (three positive samples) and for at least two sampling methods (≤ 2 positive samples). NPS, nasopharyngeal swab; NTMS, nasal midturbinate swab; SALIVA, saliva.

Previous studies reported that the sensitivity of the SARS-Cov-2 detection by RT-qPCR in saliva samples was 69.2–100% compared with that of initial diagnosis in throat and NPSs from hospitalized patients (9, 18–24). However, our data are valuable since all sampling methods provided from the same patients and our study included positive and negative samples for SARS-Cov-2. Through this study design, we eliminated the difference of sensitivity that can surge between the different sampling methods due to differences in the clinical background of patients and in the sample collection, because all samples are collected in the same patient and at the same time. Our data revealed differences in the sensitivity of SARS-Cov-2 detection obtained for the three sampling methods. Saliva sampling allowed to detect all cases with Ct values <33 (Figure 3) suggesting that the saliva sample might be a promising alternative method for COVID-19 diagnosis. It has been reported that Ct values > 35 correlates with a lesser infectivity of SARS-Cov-2 in cell culture (25). In addition, only one RT-qPCR kit, the Allplex^TM^ 2019-nCoV Assay Kit was used in our study for the SARS-Cov-2 detection in saliva samples. Although the Allplex^TM^ 2019-nCoV Assay Kit provided valuable results, this fact did not exclude that other RT-qPCR kits could improve the sensibility of the detection in this kind of sample. In effect, it previously showed a wide variability in the sensitivity of RT-qPCR solutions for SARS-CoV-2 detection, principally due to a component difference of RT-qPCR kits (buffers, enzymes, and reagent contents in general) (26).

Our data also suggest that neither a single NPS nor a single saliva specimen is not 100% sensitive for the detection of COVID-19. This is consistent with previous literature (10), emphasizing that a single negative test does not rule out disease in patients with a high pre-test probability of COVID-19. To elude this issue, it should be considered repeated sampling and/or collected various sample types in the same patient for improving the yield. For example, among patients with a high pre-test probability for COVID-19 and a negative NPS swab, repeating the NPS swab and also collecting a saliva sample could be considered. In addition, saliva sample has clinical advantages because the sampling method is non-invasive and can be collected by patients themselves. However, a self-sample collection of saliva required that patients receive previously clear instructions. Just asking patients to spit a teaspoon of saliva into a specimen container can lead to not providing a full teaspoon of saliva, which can then affect the sensitivity of the COVID-19 diagnostic. To avoid this problem, saliva samples of our study were collected according to an *in-house*-validated protocol from which nursing staff assessed the collection of saliva samples. Other studies testing saliva samples for COVID-19 diagnostics used self-collected saliva samples, which cannot ensure data accuracy. Without clear instruction and training, patients can contribute to the increased risks of sample contamination and decrease the quality of collected samples that can affect the pathogen detection (false negatives and positives) and then the diagnostic.

Also, the collection time of saliva samples appears to be an important factor for the detection of SARS-Cov-2. At the 1st week of illness, when viral concentrations have been reported to be highest, it was showed that the sensitivities of saliva samples, as well as those of NPS, were the highest and differed just by 6%. When the samples were collected during the 2nd week of illness or later, this difference of sensitivity between both sampling methods was increased by 20% (27). The saliva flow rate might be involved in this phenomenon, because it could differ according to the advance of the disease and then affect the viral load in the saliva. It is therefore important to consider the time of illness for the determination of sampling methods; NPS samples seem more sensitive than saliva for SARS-CoV-2 detection during the later illness.

It is important to highlight that the analysis of saliva samples also shows several disadvantages. In comparison with others such as nasopharyngeal and nasal samples, saliva samples present a higher risk of spillage. To avoid this problem, saliva collection devices are available. However, the use of this equipment increases the cost of the clinical test, risen by close to 30%. In general, sampling kits such as those for nasopharyngeal and nasal samples include swabs and transport medium, in contrast to sampling saliva. There are few specialized devices for saliva samples that include transport media to preserve viral RNA saliva samples, and they are more expensive compared to the NPS sampling kits. The cost of clinical diagnosis remains an important point, because not all health insurance programs can support it. For example, although the Chilean State subsidizes the provision of PCR test, through the “Fondo Nacional de Salud” (FONASA), it does not include the supplementary devices resulting from the saliva sample process. For this reason, it is sometimes necessary to prepare wide-mouth containers with viral transport media for saliva sampling. Unlike to others, because they are more viscous, saliva samples require a pre-treatment step, with proteinase K spending more time in processing.

In conclusion, NPS remains the optimal sample for COVID-19 diagnosis. Although saliva samples have a lower analytical sensitivity, they represent a promising alternative sampling method if the NPS cannot be considered. In addition, it should be interesting in the future to evaluate other RT-qPCR kits, because it could improve the detection of SARS-Cov-2 in saliva samples.

## Data Availability Statement

The original contributions generated for this study are included in the article/supplementary material, further inquiries can be directed to the corresponding author/s.

## Ethics Statement

The studies involving human participants were reviewed and approved by Ethics Committee of Clínica Dávila. The patients/participants provided their written informed consent to participate in this study.

## Author Contributions

CT and MF contributed to conception and design, data analysis, and drafted the manuscript. CM, MI, PN, ML, GC, and AV contributed to collect and process the samples. IP contributed to data interpretation and critically revised the manuscript. All authors gave final approval and agree to be accountable for all aspects of the work.

## Conflict of Interest

The authors declare that the research was conducted in the absence of any commercial or financial relationships that could be construed as a potential conflict of interest.
